# Prevalence and related risk factors of chronic kidney disease among adults in Luxembourg: evidence from the observation of cardiovascular risk factors (ORISCAV-LUX) study

**DOI:** 10.1186/s12882-017-0772-6

**Published:** 2017-12-08

**Authors:** Ala’a Alkerwi, Nicolas Sauvageot, Illiasse El Bahi, Charles Delagardelle, Jean Beissel, Stephanie Noppe, Paul J. Roderick, Jennifer S. Mindell, Saverio Stranges

**Affiliations:** 1 Department of Population Health, Epidemiology and Public Health Research Unit, Luxembourg Institute of Health (LIH) (formerly CRP-Santé), Grand-Duchy of Luxembourg, 1A-B, rue Thomas Edison, L-1445 Strassen, Luxembourg; 2 Service of Cardiology, Centre Hospitalier du Luxembourg, Grand-Duchy of Luxembourg, Luxembourg, Luxembourg; 3 Academic Unit of Primary Care and Population Sciences, Faculty of Medicine, University of Southampton, Southampton General Hospital, South Academic Block, Tremona Road, Hampshire, Southampton, SO16 6YD UK; 4 Research Department of Epidemiology & Public Health, UCL (University College London), London, WC1E 6BT UK; 5 Department of Epidemiology and Biostatistics, Schulich School of Medicine & Dentistry, Western University, London, Ontario Canada; 6 Department of Family Medicine, Schulich School of Medicine & Dentistry, Western University, London, Ontario Canada

**Keywords:** Chronic kidney disease (CKD), Glomerular filtration rate, Albuminuria, Population-based study, Epidemiology

## Abstract

**Background:**

Evidence on stages of renal impairment and related risk factors in Luxembourg is lacking. This study aimed to assess the prevalence of chronic kidney disease (CKD) and identify potential correlates among the general population, using the recent definition suggested by the Kidney Disease Improving Global Outcomes guidelines.

**Methods:**

Data analysed from 1361 participants aged 18–69 years, enrolled in the Observation of Cardiovascular Risk Factors in Luxembourg (ORISCAV-LUX) study, 2007–08. Descriptive and multivariable logistic regression analyses were performed to identify demographic, socio-economic, behavioural, and clinical factors associated with CKD, defined as a single estimated glomerular filtration rate (eGFR) measure <60 ml/min/1.73m^2^ and/or urinary albumin: creatinine ratio (ACR) > 30 mg/g.

**Results:**

Overall, 6.3% had CKD, including 4.4% and 0.7% with moderate and severe macroalbuminuria respectively. 0.1% had kidney failure (eGFR **<** 15 ml/min/1.73 m^2^). CKD was higher among subjects with primary education and risk increased significantly with age; the odd ratio was more than 2-fold higher among participants aged 50–69 years. Hypertension and diabetes were associated with more than 3-fold and 4-fold higher risks of CKD [adjusted odd ratio (AOR 3.46 (95%CI 1.92, 6.24), *P* < 0.001] and [AOR 4.45 (2.18, 9.07), P < 0.001] respectively. Increased physical activity measured as total MET-hour/week was independently associated with a lower odds of CKD (*P* = 0.035).

**Conclusion:**

The national baseline prevalence estimate of CKD, a neglected public health problem, stresses the benefit of early detection particularly in high-risk subjects with associated cardiovascular pathologies (e.g. hypertension, diabetes), to prevent and defray costs related to eventual complications.

## Background

During the last decade, there has been a rising interest in the epidemiology of chronic kidney disease (CKD), which is now recognized as an important public health problem worldwide [1]. Patients with CKD are at high risk for progression to end stage renal disease (ESRD); a condition often requiring costly renal replacement therapy in the form of dialysis or transplantation. Although over 2 million people now require chronic renal replacement therapy worldwide [2, 3], only a minority of patients who are at risk for developing ESRD are under medical attention [4]. Moreover, CKD is associated with eight- to ten-fold increased risk of cardiovascular disease (CVD) mortality [5, 6]. Other complications include acute kidney injury, increased risk of infection, cognitive decline, anaemia, mineral and bone disorders and fractures [7].

The economic impact of CKD is enormous, whether related to direct healthcare cost or to indirect productivity lost with profound consequences on the quality of life of the individual, his family and society [3]. Recently, the Global Burden of Diseases, Injuries, and Risk Factors Study (GBD) ranked low glomerular filtration rate (GFR) as the 12th leading risk factor for death at the global level, and the 14th risk factor for Disability-Adjusted Life-Years (DALYs) among 79 risk factors in 2013 [8]. In the past decade, attention has moved from *treating* only advanced stages of CKD toward *prevention* at earlier stages of CKD [4]. However, due to the asymptomatic nature of slowly progressing renal damage, CKD is frequently undetected until the very late stages, with few opportunities for prevention. Therefore, focusing efforts on early detection and treatment of CKD can prevent or delay progress to kidney failure or other adverse outcomes [9].

Low GFR has been identified among the top 10 leading cause of DALYs for both sexes in Luxembourg [10], where cardiovascular mortality accounts for about one-third of all deaths [11], and associated cardio metabolic pathologies, such as diabetes, hypertension, lipid disorders and obesity are demonstrably high [12, 13]. The objectives of this study were to provide baseline evidence on the prevalence of CKD among the general population in Luxembourg, to assess the different stages of CKD and identify potential socio-economic, clinical and behavioural correlates. Accurate assessment of CKD stages among the general population may provide important evidence-based information for policy makers and healthcare professionals regarding strategies for CKD prevention and healthcare planning.

## Methods

### Study design and participants

Analyses were based on data from the Observation of Cardiovascular Risk Factors in Luxembourg (ORISCAV-LUX) survey, a nationwide population-based cross-sectional study of adults in Luxembourg. A comprehensive description of the study design, sampling method, sample representativeness and data collection have been previously reported [12, 14]. Briefly, a stratified random sample of 1432 participants, aged 18–69 years, was recruited between November 2007 and January 2009, selected from the national insurance registry. For the present study, data from 1359 subjects were available for analyses, as 73 (5.1%) were excluded, because of missing blood samples to evaluate renal function.

### Data collection and measurements

Participants self-reported their health status, socioeconomic status, personal and family medical histories and medication use. Information concerning their lifestyle factors (cigarette smoking, alcohol intake and physical activity) was collected by using Fagerström Test, Alcohol Use Disorder Test (AUDIT) and International Physical Activity Questionnaire (IPAQ), respectively. The IPAQ allowed to calculate total metabolic equivalent (METmin/week) for walking, moderate and vigorous physical activities [15]. The validated semi-quantitative FFQ [16, 17] was use to collect data on dietary habits. Participants recorded the frequency of consumption and portion size of 134 foods and beverages.

The clinical examination included measurements of height, weight, and blood pressure (BP). Systolic BP and diastolic BP (in mm Hg) were measured at least three times with a minimum 5-min interval, by using Omrom® MX3 plus automated oscillometric Blood Pressure Monitor (O-HEM-742-E; Matsusaka, Japan). Participants were classified as hypertensive when the mean of the two last measurements was ≥140 mmHg systolic or ≥90 mmHg diastolic or when they reported taking antihypertensive medications.

Height and weight were measured by using a digital column scale (Seca 701; Hamburg Germany). BMI was calculated as weight in kg divided by height in m^2^. Waist circumference (WC, cm) was measured at the level midway between the twelfth rib and the uppermost lateral border of the iliac crest during normal expiration. Obesity was defined as BMI ≥30 Kg/m^2^.

Blood samples were drawn after an overnight 8-h fast and analysed at the national laboratory. Several parameters were assessed including liver enzymes (aspartate aminotransferase AST, alanine transaminase ALT and gammaglutamyl transpeptidase γGT, all in mg/dl), lipids (total cholesterol, triglycerides, high-density lipoprotein HDL and low-density lipoprotein LDL, all in mg/dl), glycaemic biomarkers (fasting plasma sugar FPG in mg/dl and haemoglobin A1c in %), high sensitivity C-reactive protein (hs-CRP; μg/l) and serum uric acid and creatinine (Cr) concentrations in mg/dl. For the assessment of Cr, an enzymatic method by Roche on a Cobas c501 instrument was used, which has calibration traceable to an isotope dilution mass spectrometry (IDMS) reference measurement procedure.

Renal function was evaluated by using estimated GFR (eGFR), based on the widely used 4-variable Modification of Diet in Renal Disease Study (MDRD) equation, as follows: eGFR = 175* (serum Cr (mg/dl))^-1.154^ * age^-0.203^ * (0.742 for women)* 1.21 if black, where GFR is expressed as ml/min/1.73 m^2^ of body surface area, age in years and serum Cr is expressed in mg/dl [1, 18]. Then, eGFR were classified into stages 1–5, with stages 1 and 2 requiring the presence of kidney damage such as proteinuria as well as reduced eGFR to identify CKD [19].

Urine samples were collected as early morning, mid-stream urine specimens. Urinary albumin was assessed using the immunoturbidimetric assay Tinaquant Albumin Gen. 2 by Roche Diagnostics (Mannheim, Germany). Albumin: creatinine ratio (ACR in mg of albumin/g of creatinine) was determined as an early marker of glomerular injury and kidney damage. Abnormal levels were divided into macroalbuminuria (ACR >300 mg/g Cr) [20], which indicates advanced kidney disease and microalbuminuria (ACR ≥30 mg/g and <300 mg/g Cr) [19, 21], which indicates early-stage kidney disease, whereas ACR level of 0–29 mg/g Cr indicates non-clinically detected microalbuminuria.

### CKD and staging

In the present study, subjects were classified as having CKD when eGFR was <60 ml/min/1.73m^2^ (G3-G5), and/or ACR >30 mg/g Cr (level A2-A3) according to the Kidney Disease Improving Global Outcomes (KDIGO) guidelines [22, 23].

To permit an international comparison, we also presented the prevalence by using the Chronic Kidney Disease Epidemiology Collaboration (CKD-EPI) equation as follows: GFR = 141 × min (s-Cr /κ, 1)^α^ × max(s-Cr /κ, 1)^-1.209^ × 0.993^Age^ × 1.018 [if female] × 1.159 [if black] [24], where: s-Cr is serum creatinine in mg/dl, κ is 0.7 for females and 0.9 for males, α is −0.329 for females and −0.411 for males, min indicates the minimum of s-Cr/κ or 1, andmax indicates the maximum of s-Cr/κ or 1.

### Statistical analysis

All statistical analyses were performed using the Statistical Package for the Social Sciences SPSS Statistics, Inc. version 24.0 software. Results were considered to be significant at the 5% level (*P* < 0.05). Initially, the prevalence of different stages of CKD were estimated and expressed as counts and proportions (%), by using both MDRD and CKD-EPI equations. To account for the stratified random sampling method, weighted statistical methods were applied to produce nationally representative CKD prevalence estimates. A sampling weight equal to the inverse probability of unit selection was allocated to each subject from the same stratum [14].

Then, descriptive analyses were performed to present demographic, socio-economic, behavioral, and cardio metabolic risk factors of participants with CKD, by using MDRD equation. Univariate logistic regression analyses were applied to identify the factors significantly associated with CKD prevalence. Results were expressed in terms of odds ratio (OR) and the respective 95% CI. For categorical variables, ‘low risk’ participants (younger age, women, Luxembourgers, tertiary level of education, living above poverty threshold, non-smokers, non-drinkers, physically active, absence of obesity, diabetes or hypertension, and without family history of selected medical conditions) were taken as reference categories.

Next, multivariable logistic regression analyses were performed to identify the independent contribution of socio-demographic, behavioral and biological factors to the risk of having CKD estimated by MDRD equation. Based on a literature review and on statistical criteria (variables showing *P* < 0.05 in the univariate analyses), the following variables were introduced in the multivariable model: age groups (18-49y; 50-69y), sex, education level (primary; secondary; tertiary), BMI, WC, HDL-cholesterol, serum triglycerides, serum ALT, hypertension (yes; no), diabetes (yes; no), and MET-hour/week for physical activity. To take account of medication intake, diabetes and hypertension were chosen rather than individual biomarkers such as FPG, HbA_1_c, systolic and diastolic BP. Obesity (yes; no) was not included in the multivariable model to avoid over-adjustment [25], likewise serum Cr as this variable is part of the eGFR formula. Concerning race, this “mainly Europid” study is underpowered to examine racial differences.

## Results

### Prevalence of CKD

Table 1 shows that 6.3% (89 participants) had CKD, where the eGFR estimated by MDRD, including 4.4% and 0.7% with moderate and severe albuminuria respectively, and 0.1% with kidney failure (eGFR **<** 15 mL/min/1.73 m^2^).

**Table 1:** Identification of CKD estimated by MDRD, according to levels of eGFR and ACR categories, among 1359 participants in the Observation of Cardiovascular Risk Factors in Luxembourg (ORISCAV-LUX) study, 2007-08, aged 18–69 years

eGFR and ACR categories and risk of adverse outcomes			ACR categories (mg/g Cr), description and range			
			<30	30–300	>300	
			Normal to mildly increased	Moderately increased	Severely increased	
			Normoalbuminuria	Microalbuminuria	Macroalbuminuria	
			A1	A2	A3	Total
GFR categories (ml/min/1.73 m^2^)	≥90 Normal and high	G1	612 (48.4%) [137,188]	37 (2.8%) [7919]	5 (0.3%) [940]	654 (51.5%) [146,047]
	60–89 Mild reduction	G2	658 (45.3%) [128,547]	23 (1.6%) [4647]	2 (0.1%) [356]	683 (47.1%) [133,550]
	30–59.9 Moderate impairment	G3	17 (1.1%) [3125]	0	2 (0.1%) [348]	19 (1.2%) [3473]
	15–29.9 Severe impairment	G4	1 (0.1%) [194]	0	1 (0.1%) [222]	2 (0.1%) [416]
	<15 Kidney failure	G5	0	0	1 (0.1%) [165]	1 (0.1%) [165]
		Total	1288 (94.9%) [269,054]	60 (4.4%) [12,566]	11 (0.7%) [2032]	1359 (100%) [283,652]

Data represent number (%) of participants having the pathology [Estimated population in Luxembourg]. Sample weighting used to present data

*ACR* albumin: creatinine ratio, *CKD* chronic kidney disease, *GFR* glomerular filtration rate

CKD identified in people with GFR <60 ml/min/1.73 m2 (GFR categories G3-G5) or markers of kidney damage. In the absence of evidence of kidney damage such as albuminuria, neither eGFR category G1 nor G2 fulfil the criteria for CKD

By using CKD-EPI equation, the prevalence estimate was similar (5.9%; 82 subjects), with a difference of 7 cases which were not detected via this definition (Table 2).

**Table 2:** Identification of CKD estimated by CKD-EPI, according to levels of eGFR and ACR categories, among 1359 participants in the Observation of Cardiovascular Risk Factors in Luxembourg (ORISCAV-LUX) study, 2007-08, aged 18–69 years

eGFR and ACR categories and risk of adverse outcomes			ACR categories (mg/g Cr), description and range			
			<30	30–300	>300	
			Normal to mildly increased	Moderately increased	Severely increased	
			Normoalbuminuria	Microalbuminuria	Macroalbuminuria	
			A1	A2	A3	Total
GFR categories (ml/min/1.73 m^2^)	≥90 Normal and high	G1	923 (70.5%) [199,875]	48 (3.5%) [9983]	6 (0.4%) [1119]	977 (74.4%) [210,978]
	60–89 Mild reduction	G2	354 (23.6%) [67,172]	12 (0.9%) [2582]	1 (0.1%) [178]	367 (24.7%) [69,933]
	30–59.9 Moderate impairment	G3	10 (0.6%) [1812]	0	2 (0.1%) [348]	12 (0.8%) [2161]
	15–29.9 Severe impairment	G4	1 (0.1%) [194]	0	1 (0.1%) [222]	2 (0.1%) [416]
	<15 Kidney failure	G5	0	0	1 (0.1%) [165]	1 (0.1%) [165]
		Total	1288 (94.9%) [269,054]	60 (4.4%) [12,566]	11 (0.7%) [2032]	1359 (100%) [283,652]

Data represent number (%) of participants having the pathology [Estimated population in Luxembourg]. Sample weighting used to present data

*ACR* albumin: creatinine ratio, *CKD* chronic kidney disease, *GFR* glomerular filtration rate

CKD identified in people with GFR <60 ml/min/1.73 m2 (GFR categories G3-G5) or markers of kidney damage. In the absence of evidence of kidney damage such as albuminuria, neither eGFR category G1 nor G2 fulfil the criteria for CKD

### Factors associated with CKD

Table 3 outlines the associations between a wide-range of demographic, behavioral and clinical characteristics of individuals with CKD, using the MDRD definition. Overall, the prevalence of CKD increased significantly with age; the odds ratio increased more than 2-fold among subjects aged 50–69 years. The distribution of CKD varied significantly according to education level; the frequency of CKD was higher among subjects with primary education. There was no significant difference according to geographical district or country of birth. Among lifestyle factors, increased physical activity in MET-hour/week was associated with slightly, but statistically significantly, lower risk [odds ratio (95%CI): 0.97 (0.95, 0.99); *P* = 0.01)].

**Table 3:** Distribution of CKD according to demographic, socio-economic, behavioural and health-related characteristics of participants^a^ in ORISCAV-LUX study, 2007-08, aged 18–69 years

Characteristics	n	No CKD	CKD^b^	Crude OR	95% CI	*P*-value
Socio-demographic characteristics						
Age (years) Mean (SD)	1359	43.8 ± 0.36	49.47 ± 1.60	1.03	(1.02–1.05)	<0.001
	1359	N (%)	N (%)			
18–49		855 (67.3)	41 (46.1)	1 (Ref.)		<0.001
50–69		415 (32.7)	48 (53.9)	2.4	(1.56–3.72)	
Sex	1359					0.88
Female		652 (51.3)	45 (50.6)	1(Ref.)		
Male		618 (48.7)	44 (49.4)	1.03	(0.67–1.58)	
Race	1359					0.42
White		1264 (99.5%)	88 (98.9%)	1(Ref.)		
Black		6 (0.5%)	1 (1.1%)	0.41	(0.05–3.51)	
Education level	1345					0.02
Tertiary		329 (26.2)	21 (23.6)	1(Ref.)		
Secondary		599 (47.7)	33 (37.1)	0.86	(0.49–1.51)	
Primary		328 (26.1)	35 (39.3)	1.67	(0.95–2.93)	
Country of birth	1359					0.38
Luxembourg		762 (60)	53 (59.6)	1(Ref.)		
Portugal		147 (11.6)	15 (16.9)	1.47	(0.80–2.67)	
Other European countries		77 (6.1)	3 (3.4)	0.91	(0.52–1.58)	
Non-European countries		284 (22.4)	18 (20.2)	0.56	(0.17–1.83)	
Geographical district	1359					0.17
Luxembourg		926 (72.9)	72 (80.9)	1(Ref.)		
Diekirch		190 (15)	7 (7.9)	0.47	(0.21–1.05)	
Grevenmacher		154 (12.1)	10 (11.2)	0.83	(0.42–1.65)	
Poverty threshold	1176					0.29
Above		236 (21.4)	19 (26.8)	1(Ref.)		
Below		869 (78.6)	52 (73.2)	1.34	(0.78–2.32)	
Lifestyle factors						
Alcohol consumption	1359					0.22
Non-drinkers		182 (14.3)	17 (19.1)	1(Ref.)		
Drinkers		1088 (85.7)	72 (80.9)	0.71	(0.41–1.23)	
Smoking status	1359					0.69
Non-smokers		990 (78)	71 (79.8)	1(Ref.)		
Current smokers		280 (22)	18 (20.2)	0.9	(0.53–1.53)	
Total MET-hour/week, Mean (SD)	1294	13.62 (0.31)	10.58 (0.94)	0.97	(0.95–0.99)	0.01
Biochemical and clinical measurements						
		Mean (SD)	Mean (SD)			
BMI (Kg/m^2^)	1358	26.48 (0.14)	28.84 ± 0.66	1.09	(1.04–1.13)	<0.001
Waist circumference, cm	1358	89.47 (0.39)	96.19 ± 1.82	1.03	(1.02–1.05)	<0.001
Blood pressure, mm Hg						
Systolic	1357	129.08 ± 0.48	138.79 ± 2.42	1.03	(1.02–1.04)	<0.001
Diastolic	1359	82.05 ± 0.3	86.32 ± 1.39	1.03	(1.02–1.05)	<0.001
Pulse rate, beats/min	1359	68.87 ± 0.28	70.89 ± 0.99	1.02	(0.99–1.04)	0.07
FPG, mg/dl	1330	93.89 ± 0.44	110.43 ± 4.39	1.03	(1.02–1.03)	<0.001
HbA1c, %	1330	5.55 ± 0.01	6.02 ± 0.11	2.81	(2.08–3.8)	<0.001
Serum total cholesterol, mg/dl	1331	201.35 ± 1.13	196.78 ± 5.51	1.00	(0.99–1.00)	0.31
Serum HDL cholesterol, mg/dl	1331	61.49 ± 0.48	54.51 ± 1.86	0.97	(0.96–0.99)	<0.001
Serum LDL cholesterol, mg/dl	1331	124.36 ± 0.98	121.4 ± 4.47	0.998	(0.99–1.00)	0.45
Serum triglycerides, mg/dl	1331	113.07 ± 2.43	162.89 ± 18.81	1.00	(1.00–1.00)	<0.001
Serum haemoglobin, mg/dl	1358	14.68 ± 0.037	14.65 ± 0.17	0.98	(0.84–1.15)	0.83
Serum creatinine, mg/dl	1359	0.83 ± 0.004	0.94 ± 0.047	10.45	(3.47–31.45)	<0.001
Serum uric acid, mg/dl	1359	5.08 ± 0.038	5.31 ± 0.17	1.12	(0.97–1.31)	0.13
hs-CRP, μg/l	1359	2.65 ± 0.13	3.25 ± 0.44	1.02	(0.98–1.05)	0.26
Serum ALT, mg/dl	1359	25.39 ± 0.45	29.56 ± 1.82	1.01	(1.00–1.02)	0.02
Serum AST, mg/dl	1359	22.54 ± 0.25	23.1 ± 0.91	1.01	(0.98–1.03)	0.56
Serum γGT, mg/dl	1359	32.94 ± 1.22	37.18 ± 3.17	1.00	(0.99–0.01)	0.37
Associated pathologies						
		N (%)	N (%)			
Hypertension^d^	1358					<0.001
No		816 (64.3)	26 (29.2)	1(Ref.)		
Yes		453 (35.7)	63 (70.8)	4.36	(2.72–6.99)	
Family history of HTA	1145					0.93
No		645 (60.3)	44 (58.7)	1(Ref.)		
Yes		425 (39.7)	31 (41.3)	1.07	(0.66–1.72)	
Diabetes mellitus^c^	1331					<0.001
No		1196 (96.3)	68 (77.4)	1(Ref.)		
Yes		46 (3.7)	21 (23.6)	8.03	(4.54–14.21)	
Family history of diabetes	1287					0.06
No		938 (78)	58 (69)	1(Ref.)		
Yes		265 (22)	26 (31)	1.59	(0.98–2.57)	
Obesity	1358					<0.001
BMI <30 Kg/m^2^		990 (78)	49 (55.1)	1(Ref.)		
BMI ≥30 Kg/m^2^		279 (22)	40 (44.9)	2.9	(1.87–4.49)	
Dietary consumption						
		Mean (SD)	Mean (SD)			
Salt, mg/day	1270	8.91 ± 0.11	8.79 ± 0.42	0.99	(0.94–1.05)	0.81
Meat, mg/day	1282	109.16 ± 2.44	112.84 ± 8.38	1.00	(0.99–1.00)	0.7
Fruit & vegetables, g/day	1278	556.22 ± 11.47	608.34 ± 45.97	1.00	(1.00–1.00)	0.25
Animal protein, g/day	1267	62.87 ± 0.83	63.74 ± 3.23	1.00	(0.99–1.00)	0.79

*FPG* fasting plasma glucose, *HbA1c* glycated Hb, *WC* waist circumference, *ALT* Alanine transaminase, *AST* Aspartate Aminotransferase, *γ-GT* Gamma-glutamyl-transpeptidase

Data presented are means ± SE for continuous variables, otherwise numbers (%) for categorical variables

^a^Difference in the number of cases is related to missing values for several variables

^b^CKD estimated by MDRD equation

^c^Participants were classified as having diabetes when serum glucose was 126 mg/dl or when they reported taking antidiabetic medications

^d^Participants were classified as having HT when systolic blood pressure ≥ 140 mmHg and/or diastolic blood pressure ≥ 90 mmHg and/or taking antihypertensive medications

In univariate analyses, the prevalence of CKD differed substantially according to biochemical and clinical measurements, including BMI, WC, systolic and diastolic BP, FPG, glycated Hb, HDL-cholesterol, triglycerides and serum Cr (all *P* < 0.001). Consequently, the prevalence estimates were significantly higher in the presence of obesity, hypertension, and diabetes and respective odd ratios were increased 3-fold, 4-fold, and 8-fold respectively (all P < 0.001). Concerning dietary intake, there were no associations of CKD with salt, animal protein, meat, fruit or vegetable consumption.

In the multivariable analysis, increased physical activity remained independently correlated with lower odds of CKD [Adjusted Odds Ratio (AOR 0.97 (95%CI 0.95–0.99); *P* = 0.035)]. Hypertension and diabetes were also independently associated with more than 3-fold and 4-fold higher risk of CKD [AOR 3.46 (1.92, 6.24); *P* < 0.001] and [AOR 4.45 (2.18, 9.07); *P* < 0.001], respectively (Table 4).

**Table 4:** Independent demographic, socio-economic, behavioural and health-related correlates to CKD as identified by multivariate logistic regression; in 1256 participants in the ORISCAV-LUX study, 2007-08, aged 18–69 years

		Multivariable Analysis	
Covariates	Categories	Adjusted OR (95% CI)	*p*-value
Gender	Male vs female	0.52 (0.28–0.97)	0.041
Educational Level			0.35
	Primary v. tertiary	0.70 (0.38–1.29)	
	Secondary v. tertiary	1.01 (0.53–1.92)	
Total MET-hour/week	5 units increase	0.97 (0.95–0.99)	0.032
BMI	1 unit increase	0.95 (0.86–1.05)	0.35
WC	1 cm increase	1.01 (0.97–1.05)	0.71
HDL cholesterol	1 mg/dl increase	0.97 (0.95–0.99)	0.009
Serum triglycerides	1 mg/dl increase	1.002 (1.00–1.004)	0.02
Serum ALT	1 mg/dl increase	0.99 (0.98–1.01)	0.57
Hypertension	Hypertensive v. non-hypertensive	3.46 (1.92–6.24)	<0.001
Diabetes	Diabetic v. non-diabetic subjects	4.45 (2.18–9.07)	<0.001
Age groups	50–69 years v. 18–49 years	1.36 (0.79–2.35)	0.26

*BMI* body mass index, *WC* waist circumference, *HDL* cholesterol, high-density lipoprotein cholesterol, *ALT* Alanine transaminase

## Discussion

CKD has been recognized as a worldwide public health problem due to rising prevalence, association with CVD mortality, poor outcomes, and dramatic complications which imply high costs and important burden on health care systems [19]. Renal epidemiology has blossomed in recent years, following several publications on renal impairment diagnosis and uniform clinical practice guidelines. This is the first nationwide study to describe the epidemiology of CKD in Luxembourg, based on a standardized definition and staging of renal impairment, as suggested by the internationally accepted KDOQI guidelines [19, 22].

Our findings indicate that more than 6% of the general population in Luxembourg (representing >18,000 adults nationally) suffered from one of the 5 stages of renal impairment. Moreover, 0.7% (representing >2000 people) had severe macroalbuminuria (>300 mg/g Cr), and 0.1% had kidney failure. Normal individuals usually excrete very small amounts of protein in the urine. Persistently increased protein excretion is usually a sensitive marker of kidney damage. These findings flag a neglected public health issue in Luxembourg, which warrants national attention and further investigations.

These prevalence estimates were comparable to data from Lausanne city in Switzerland, where one in 10 adults suffers from CKD, using similar diagnostic criteria [26]. Recent CKD Burden Consortium data suggests a substantial variation (from 3.3% to 17.3%) in CKD prevalence across Europe [27]. Based on earlier studies, a South-North gradient has been observed in CKD prevalence estimates, being higher in northeastern Italy (13.2%) [28] and Galician population in Spain (12.7%) [29] compared with Iceland (age-adjusted prevalence of low eGFR for adults, aged 35–80 years, was 4.7 and 11.6% for men and women, respectively) [30]. In the USA, 25 million people (about 12%) are estimated to have CKD, whereas only <0.2% (<500,000) have kidney failure treated by dialysis or transplantation [1, 31]. Similar prevalence estimates have been reported in Australia [32], and some reports note an increasing prevalence over time [33, 34]. Although our national CKD estimates are lower than the worldwide range (8–16%), [7] their public health burden is worsening with escalating CVD mortality and comorbidities, presenting substantial medical expenses.

Thus far, studies on CKD prevalence have been hampered by selection bias, or inappropriate detection criteria to define CKD stages [35]. Direct international comparisons is difficult due to important methodological differences regarding the setting of the target population, study design, as well as to the variety of CKD definitions used across different studies.

There are a number of potential factors that may have contributed to the relatively high prevalence of CKD among adults in Luxembourg. Consistent with previous reports [26], an elevated prevalence of CKD was observed in hypertensive (70.3%), diabetic (23.1%) and obese (44%) participants in the ORISCAV-LUX study. These conditions are highly prevalent in the Luxembourg population, as supported by our previous findings from ORISCAV-LUX study population [12]. The likelihood for CKD was significantly and independently increased by 3-fold and 4-fold in the presence of hypertension and diabetes, respectively. In addition, elevated serum triglycerides was substantially associated with higher odds for CKD. Evidence suggests that hypertension and diabetes are two major causes of kidney disease [36, 37]. As a complication, high BP may also develop early during the course of CKD and is associated with adverse outcomes, in particular, faster loss of kidney function and development of CVD [19]. In addition, the risk of CVD, retinopathy, and other diabetic complications is higher in patients with diabetic kidney disease than in diabetic patients without kidney disease [19]. Recently, accumulated evidence indicates that the adverse outcomes of CKD can be prevented or delayed through therapeutic interventions during earlier stages, including blood glucose control in diabetic subjects, regular BP control, treatment with angiotensin-converting enzyme inhibitors and angiotensin-receptor blockers, and dietary protein restriction. Because of the well-known interactions between CVD, hypertension, diabetes, and CKD, these findings have important clinical and public-health implications, in targeting these “high-risk” population subgroup of the population, who may benefit most from treatment to reduce progression and delay the onset of ESRD and cardiovascular complications [3, 38].

Increased physical activity was independently associated with lower odd for CKD. This association remained significant after further adjustment for demographic, socio-economic, and other lifestyle factors. From a public health standpoint, these findings may be interesting. Only a few population-based studies have reported this relationship [39, 40]; further prospective studies are needed to replicate this finding.

### Strengths and limitations

This study is has several strong points. Our findings are based on a large nationwide, population-based sample of general adults in Luxembourg [12], showed to be comparable with the non-participants regarding demographic and clinical characteristics, hence reducing the potential selection bias [41]. Data weighting was applied to provide population-representative prevalence estimates. Kidney function was evaluated according to the most recent guidelines, based on a combination of eGFR which provides data on CKD stages, and urinary ACR which allows identification of 3 levels of microalbuminuria [4]. To estimate GFR, we applied the most widely used modified MDRD formula in clinical practice and epidemiological research, which accounts for the difference in assay methods by using correction factors [18, 42]. This formula is now being used for direct reporting of eGFR by our national laboratory in Luxembourg and other international laboratories to identify and monitor patients with reduced renal function [42]. To permit international comparison, prevalence estimates by CKD-EPI equation were presented and showed a minor difference. Additionally, the ORISCAV-LUX survey measured a large set of potential risk factors for CKD, including demographic, socio-economic, clinical, biological behavioural and dietary variables that have been rarely altogether investigated in similar studies.

Potential limitations include factors related to the cross-sectional design of the study which precludes inferences regarding causal relationships, single measures of eGFR and ACR due to unavailability of further samples to assess persistence pathology for ≥3 months; in addition to unavailability of data for participants older than 69 years, who have highest CKD prevalence estimates. Other shortcoming points include potential misclassification of albuminuria due to early- (not first-) morning sample; some statistically significant results may not be clinically significant due to low overall number of cases (89 with CKD), and extremely low numbers of person with higher-stage CKD (unstable estimates) with potential collinearity of variables.

CKD prevalence estimation is central to CKD management and prevention planning at the population level [27] and thereby help estimate the growing burden and demand for CKD services concomitant with aging population. We trust that our findings contribute to filling gaps on the worldwide atlas examining heterogeneities in CKD prevalence estimates. Such knowledge may provide an insight on recommendation to extend screening to people without diabetes or hypertension.

## Conclusions

This is the first evidence-based report on the epidemiology of CKD in Luxembourg, highlighting that early preventive measures are needed to detect chronic kidney impairment and to reduce the incidence and mortality arising from the associated comorbidities. Given the burden of this public health problem [10], such data should guide national authorities and contribute to increase the awareness of scientific societies regarding the benefit of early detection of CKD, particularly in more susceptible and more disadvantaged groups. Earlier detection and appropriate management of CKD subjects will reduce cardiovascular events and slow further deterioration in renal function in these patients. These measures altogether could defray costs related to eventual ESRD development and higher risk of cardiovascular events.

## Abbreviations

ACR: Albumin: creatinine ratio
ALT: Alanine transaminase
AST: Aspartate Aminotransferase
BMI: Body mass index
CKD: Chronic kidney disease
CKD-EPI: Chronic Kidney Disease Epidemiology Collaboration
Cr: Creatinine
CVD: Cardiovascular disease
ESRD: End stage renal disease
FPG: Fasting plasma glucose
GFR: Glomerular filtration rate
HbA1c: Glycated Haemoglobin
hs CRP: High sensitivity C- reactive protein
KDIGO: Kidney Disease Improving Global Outcomes
KDOQI: National Kidney Foundation’s Kidney Disease Outcome Quality Initiative
MDRD: Modification of Diet in Renal Disease Study
OR: Odd ratio
ORISCAV-LUX: Observation of Cardiovascular Risk Factors in Luxembourg
SPSS: Statistical Package for the Social Sciences
WC: Waist circumference
γ-GT: Gamma-glutamyl-transpeptidase
